# Exploring client messages in a therapist-guided internet intervention for alcohol use disorders – A content analysis

**DOI:** 10.1016/j.invent.2021.100483

**Published:** 2021-11-11

**Authors:** Martin Kraepelien, Heather D. Hadjistavropoulos, Anne H. Berman, Christopher Sundström

**Affiliations:** a Centre for Psychiatry Research, Department of Clinical Neuroscience, Karolinska Institutet & Stockholm Health Care Services, Region Stockholm, Sweden; b Department of Psychology, University of Regina, Saskatchewan, Canada; c Department of Psychology, Uppsala University, Uppsala, Sweden; d Department of Psychology, Stockholm University, Stockholm, Sweden

**Keywords:** Client messages, Therapist-guidance, Internet interventions, Alcohol use disorder, Content analysis

## Abstract

**Background:**

There is a growing interest in offering therapist-guided internet interventions for alcohol use disorders (AUD) in regular addiction services. Elucidating the therapeutic processes during these interventions may help improve clinical delivery. The aim of this paper was to investigate written messages from client to therapist in a therapist-guided internet intervention for AUD.

**Methods:**

Data was extracted from the therapist-guided arm (*n* = 57) of a randomized trial of internet interventions for AUD. Qualitative content analysis was used to identify distinct categories of client behaviors in written messages to therapists. Coding was deductive (applying categories from past literature) as well as inductive (identifying new categories from the data). Subsequently, exploratory correlational and regression analyses were conducted to investigate whether identified client behaviors predicted module completion and drinking outcomes. Also, client questions posed in messages to therapists were categorized separately.

**Results:**

Eleven distinct behavior categories were identified, of which the two most common were *alliance* (26.6% of total categorizations) and *identifying patterns and problem behaviors* (22.8%). *Confrontational alliance rupture* was the least common category (0.4%). One new behavior category was identified inductively – *alcohol-related setback* (4.1%). In the exploratory analyses, no categories consistently predicted module completion or drinking outcomes. Client questions were most commonly posed to improve understanding or use of program content or skills.

**Discussion:**

The behavior categories, although not predictive of module completion or outcomes, may be of use for therapists, treatment developers and health care providers as a tool for understanding therapeutic processes in internet interventions for AUD.

## Highlights

• Interest in internet interventions for alcohol use disorder is growing.
• Content analysis was applied to messages from clients to therapists in a therapist-guided intervention.
• Eleven distinct behavior categories were identified.
• The most common client behavior category was alliance.
• Categorizations may be of use for digital health care providers.

## 1 Introduction

Alcohol use remains a major global public health issue (WHO, 2018), and individuals with alcohol use disorders (AUD) account for about half of the alcohol-attributed burden of disease (Rehm et al., 2010). Fewer than one in five of those with an AUD receive help (Rehm et al., 2015), and easily accessible internet interventions may serve an important role in reducing this treatment gap (Kohn et al., 2004). Internet interventions for alcohol problems have been found effective in varying populations, and reductions in drinking tend to be larger when the intervention is guided by a therapist or coach (Riper et al., 2018). Recent trials conducted in clinical settings have found that these interventions yield effects comparable to traditional face-to-face treatment (Johansson et al., 2021; Kiluk et al., 2018), suggesting that implementation of internet interventions into regular addiction services is a logical next step (Bertholet and Cunningham, 2021).

Although many people appear to benefit from internet interventions, not all are helped. In clinical practice it is beneficial to be able to effectively differentiate those who improve with a certain treatment, from those who do not (Andersson et al., 2019; Cunningham et al., 2010; Drozd et al., 2016). A relatively untapped area of investigation in this regard is to analyze correspondence between client and therapist. This type of analysis can provide clinical insights, for example, in helping therapists understand what to expect from clients, identifying those who do not benefit from the intervention at an early stage, or providing information about what type of communication is likely to be helpful – or unhelpful – for the individual client. In face-to-face psychological treatment, such analyses are a cumbersome task, as they entail recording, transcribing and coding individual therapy sessions. However, in internet interventions, all written correspondence between therapist and client is stored on a digital platform, rendering both recording and transcription unnecessary. A few studies have conducted content analyses of correspondence between client and therapist in internet interventions for anxiety and depression, focusing on either therapist (Paxling et al., 2013; Schneider et al., 2016) or client behaviors (Soucy et al., 2018; Svartvatten et al., 2015). Among studies focused on client behaviors, Svartvatten et al. identified ten client behaviors in a depression trial, and found that the behavior categories *alliance* and *experiencing positive consequences* positively correlated with reduction in depression symptoms (Svartvatten et al., 2015). Subsequently, Soucy et al., using the behavior categories identified by Svartvatten et al., found that *experiencing positive consequences* was associated with a higher degree of therapist alliance and a reduction in anxiety symptoms (Soucy et al., 2018). A further aspect of potential significance for implementation and clinical development of guided internet interventions is to map the different types of questions clients pose to their therapist. In addition to providing insight into the therapeutic processes, this may also have positive consequences for therapist training and content development. However, to our knowledge, this has only been investigated in one previous study, where questions that clients posed to their therapists in an internet intervention for depression and anxiety were placed in four different categories: *Facilitate understanding and application of materials and techniques, Therapy process, Technical issues* and *Request for additional information outside of the program* (Soucy et al., 2019).

There are, as yet, no studies specifically investigating correspondence in guided internet interventions for alcohol problems, and therefore it is not known whether the behavior categories identified in the previous studies on depression (Soucy et al., 2018; Svartvatten et al., 2015) apply to individuals with AUD. Neither have any studies specifically examined questions clients pose to their therapists in guided internet interventions for AUD. To fill this gap, the current study analyzed written messages from clients to therapists in a randomized controlled trial of a guided internet intervention for people with AUD. The study was exploratory in nature, and the specific research questions were:

1) To what extent are the behavior categories identified in a previous study of a therapist-guided internet intervention (Svartvatten et al., 2015) present in messages from clients to therapists in a guided internet intervention for AUD?
2) Are there additional AUD-related behavior categories present in the messages from clients to therapists?
3) Is the frequency of the behavior categories observed associated with adherence (module completion) and/or drinking outcomes (drinks preceding week/heavy drinking days preceding week)?
4) Applying the question categories identified in a previous study of a therapist-guided internet intervention (Soucy et al., 2019), what is the proportion of these categories posed by clients in their messages to their therapists?

## 2 Materials and methods

### 2.1 Participants

The data in the current study was collected from a previously published three-armed randomized controlled trial (Sundström et al., 2020). Participants in this trial were mostly recruited through online ads, with an inclusion process consisting of online questionnaires and a subsequent telephone interview. To be included in the randomized trial, participants had to report ≥11 (women)/≥14 (men) drinks or more in the preceding week, a score of ≥6 (women)/≥8 (men) on the Alcohol Use Disorder Identification Test (AUDIT) (Saunders et al., 1993) administered online, and SCID-5 AUD (First et al., 2015) established in a telephone interview. The study was pre-registered (ClinicalTrials.gov registration ID: NCT02645721), and was approved by the Regional Ethics Review Board in Stockholm County, Sweden (2015/2014-31, approved addendum by the Swedish Ethical Review Authority 2019-02169). All clients included in the trial provided their digital informed consent form and were then randomized to a therapist-guided internet intervention, an unguided internet intervention or a waitlist. Those randomized to the guided internet intervention (*n* = 71) were offered contact with a therapist throughout the 12-week treatment; all contact occurred through a secure built-in message system on the platform where the intervention was delivered. For the current study, only clients randomized to the guided internet intervention who wrote at least two messages to their therapist during the 12-week treatment were included (*n* = 57).

### 2.2 Intervention

Clients in the guided condition were offered the *ePlus* internet intervention, which was based on cognitive behavior therapy and relapse prevention. The intervention consisted of 13 modules, with each module devoted to a specific theme; e.g., “How to deal with craving”, “How to say no to alcohol”, “Problem solving” (Sundström et al., 2017). For each new module, clients were instructed to complete homework assignments on digital worksheets within the treatment platform, after which the therapist granted access to the next module. Therapists logged in 2–3 times per week to give feedback and encourage clients to work on the program. Ten therapists were involved in the study; six were licensed clinical psychologists, and four were clinical psychology students at the master's level. To ensure treatment integrity and adherence to the protocol, supervision of therapists was provided weekly or bi-weekly.

### 2.3 Outcome measures

The main outcome of the original trial was the number of standard drinks (12 g of ethanol) consumed during the preceding week (Sundström et al., 2020). Two primary outcomes were calculated: 1) number of drinks in the preceding week and 2) number of heavy drinking days (HDD) in the preceding week, defined as 5 or more (men) or 4 or more (women) standard drinks on a single day.

### 2.4 Coding procedures of client messages

A content analysis approach was used to code the client behaviors in the written messages sent to therapists. A directed approach was applied, allowing both deductive and inductive categories to be identified (Hsieh and Shannon, 2005). The specific procedure involved two of the authors (MK and CS) first jointly coding messages from six clients. The ten categories presented in the study by Svartvatten et al. (2015) were used as deductive categories. Given the lack of previously published studies on this topic in the AUD population, the coders also considered possible inductive categories when coding the text. After coding messages from the first six clients in dialogue, the coders slightly adjusted the ten original categories to align the category names and definitions with the messages from clients in the current trial, which concerned alcohol misuse rather than depression. The coders identified one inductive category, *Observes alcohol-related setback*. Codes were usually 1–3 sentences long, but could be longer in some cases. A preliminary coding guide with a formal definition and a list of examples of each category was created, with the purpose of making the categories as clear and distinguishable as possible. The preliminary coding guide was shared and discussed with two experts in the field (authors AHB and HH), after which a final coding guide was developed. Three additional clients were then coded jointly by MK and CS using the final coding guide. Then, six additional clients were coded separately by authors MK and CS to ascertain inter-rater reliability. After inter-rater reliability had been deemed sufficient (κ = 0.62; translates to a strength of agreement traditionally labeled as “good” (Brennan and Silman, 1992)), the remaining 42 clients were divided equally among the two authors and coded individually. All questions from clients to therapists were identified in the written messages, and then categorized according to the four categories from the study by Soucy Soucy et al. (2019): 1) Questions about facilitating understanding and application of core techniques, 2) Questions about the therapy process, 3) Questions about technical issues and 4) Questions about requesting assistance outside the scope of the program. Questions could be “double-coded”, i.e. could be categorized both as a question and as one of the behavior categories.

### 2.5 Statistical analysis

First, descriptive statistics were used to describe demographics of the clients. Second, all correspondence was entered into NVivo qualitative data analysis software (QSR International PTY Ltd., version, release 1.4.1, 2021). The frequency of statement categories was calculated by indicating how often each client exhibited statements coded as belonging to a category. Inter-rater reliability between authors MK and CS was calculated with Cohen's Kappa (κ), applying procedures outlined in NVivo10. Cohen's Kappa accounts for agreement that can arise from random chance, and a weighted Kappa coefficient was calculated to control for differences. The NVivo10 word frequency query function was used to calculate the total frequency of categories for each client, generating a frequency matrix of the categories among all clients. After this, Spearman's rank-order correlation coefficients were used to examine intercorrelations among categories, as well as to assess the correlation between categories and module completion, and residualized change scores in drinking outcomes (drinks preceding week/heavy drinking days preceding week) respectively. Residualized change scores were calculated to account for variations in pre-treatment alcohol consumption. In order to control for the total number of codes we also performed an additional hierarchical regression analysis. In this analysis, after controlling for initial drinking and total number of client behaviors coded, we examined the extent to which the client behavior categories and number of modules completed predicted drinking outcomes at post treatment or at the 3-month follow-up. We used *p* = 0.05 as the analyses were exploratory and we did not want to rule out potential relationships. All statistical calculations in the quantitative analysis were conducted using the statistical software programs SPSS 25 (IBM Corp) and R (R Core team).

## 3 Results

### 3.1 Demographics and treatment outcomes

Baseline demographic and clinical data of clients in the current analysis are presented in Table 1. After treatment, mean alcohol consumption among the 57 clients included was 9.87 (*SD* = 11.64; 95% *CI* 6.63–13.11) drinks preceding week, and 0.96 (*SD* = 1.45; 95% *CI* 0.56–1.37) heavy drinking days preceding week. At the 3-month follow-up, mean alcohol consumption was 16.81 (*SD* = 14.83; 95% *CI* 12.55–21.07) drinks preceding week, and 1.71 (*SD* = 1.96; 95% *CI* 1.15–2.28) heavy drinking days preceding week. The mean number of modules accessed was 9.44 (*SD* = 2.98) out of 13. Please see the original trial for more clinical data (Sundström et al., 2020).

**Table 1:** Baseline characteristics of clients who received therapist-guided internet intervention and sent more than one message to their therapist.

*n* = 57	Mean	SD
Age (years)	52.1	11.5
Drinks preceding week	34.2	17.1
Heavy drinking days preceding week	3.96	1.82
AUDIT	23.4	5.1


	n	%
Male	26	45.6

Highest educational level		
University	34	59.6
High school	21	36.8
Basic education	2	3.5

Marital status		
Married/partner	41	71.9
Divorced/widowed	6	10.5
Single	10	17.5

Alcohol use disorder		
Mild	1	1.8
Moderate	11	19.3
Severe	45	78.9

Previously sought alcohol treatment	16	28.1

### 3.2 Coding categories

The coders reused the categories from the study by Svartvatten et al., but adapted the categories during the coding process to the AUD population (see Table 2 for changes in categories).

**Table 2:** Coding manual used for coding message content.

Category name for the current study on AUD (category name from Svartvatten et al. study on depression)	Definition from Svartvatten et al. study on depression	Definition for the current study on AUD	Representative example quote	Number of coded categories, *n* = 944 (%)	Number of clients with coded category, *n* = 57 (%)
Alliance	Text expressing the participant's emotional ties in relation to treatment goals, the exercises or the therapist	No change	*I am so happy that you see progress in me. My level of motivation increases enormously from that!*	251 (26.6%)	51 (89.5%)
Plans to attempt behavior change or use treatment exercise (Chooses alternative behavior)	Text regarding thoughts about implementing a future alternative behavior or treatment exercise, or alternatively, a text regarding plans that the person made regarding an alternative behavior or treatment exercise	Text regarding thoughts about, or plans regarding, implementing a future alcohol related or other adaptive behavior change with potential consequences for the participants alcohol consumption, or thoughts about, or plans regarding, using a treatment exercise	*I am going travelling this week and I therefore choose the activity of exercising at the gym instead, on Wednesday at 6 pm I decide that I will exercise for 45* min *at the hotel gym.*	80 (8.5%)	33 (57.9%)
Reports on behavior change attempt or use of treatment exercise	Text that demonstrates that the participant has completed, or attempted to complete, a specific alternative behavior or treatment exercise	Text that demonstrates that the participant has completed or attempted to complete an alcohol related or other adaptive behavior change with potential consequences for the participants alcohol consumption, or completed or attempted to complete a treatment exercise	*I decided to head down to the restaurant, I have learned that a good plate of food can eliminate the “appetite” for alcohol.*	87 (9.2%)	35 (61.4%)
Observes positive consequences of behavior change attempt or use of treatment exercise	Text that expresses a positive change after the participant tried a specific alternative behavior or exercise in the treatment	Text that expresses a positive change after the participant tried an alcohol related or other adaptive behavior change with potential consequences for the participants alcohol consumption, or tried an exercise in the treatment	*I hereby report to you about last week's activities. I stayed in a hotel for two nights and I exercised at the gym one of the nights. I consumed no alcohol and that was so nice!*	68 (7.2%)	27 (47.4%)
Identifies patterns and problem behaviors	Text in which the participant identifies the relationship between internal and external behaviors and their effect on the participant's affective condition, alternatively, text that identifies avoidance and rumination	Text in which the participant identifies the relationship between internal and external behaviors and their effect on the participant's alcohol consumption, alternatively, text that identifies how alcohol consumption affects emotion	*Something makes me want to drink. I also crave sweets and coffee, but I can't identify any craving, like where in my body I feel it and that kind of thing. I also have trouble identifying feelings and having to describe them, hard to manage feelings. Maybe these are related.*	215 (22.8%)	43 (75.4%)
Confrontational alliance rupture (No change)	Text that indicates an emotional rupture between the participant and the goals, exercises or the therapist	No change	*I don't understand how you and this program works. Aren't you supposed to provide feedback on what I write??*	4 (0.4%)	4 (7.0%)
Maladaptive thinking or anticipation of failure	Text about the participant's depressive symptoms and their consequences without any suggested solution, or text that expresses mental problem-solving in which the result is uncertain, but contains possibilities of one or more negative outcomes	Text where the participant expresses their problems (alcohol-related or not) without any suggested solution, where the future is described as bleak or where the participant anticipates future failure, such as relapse	*I guess I'll have to try again. Unfortunately this is not the first time I decide on this and then fail.*	54 (5.7%)	15 (26.3%)
Avoidance of treatment	Text about the participant not having completed various parts of the treatment, from either a technical or a content-related aspect	Text about the participant not having been as active with the treatment as anticipated	*For several days, I have been thinking that I must* log *in and continue writing but sometimes I can have like a mental block from dealing with certain things.*	52 (5.5)	25 (43.9%)
Problems with treatment content (no change)	Text in the form of questions or clarifications from the participant regarding the content of the treatment, or, alternatively, expresses difficulties in filling out or reading the treatment material	Text that expresses difficulties in understanding the purpose or content of the treatment material	*I am having trouble understanding how to complete module 9… Can you give me some examples?*	32 (3.4)	18 (31.6%)
Problems with technology and administration	Text in which the participant expresses difficulties and problems with techniques relating to the platform, or asks for help with this, or alternatively, reports problems with the administration of the treatment.	Text that expresses technical difficulties, such as having problems logging in or understanding the platform	*I don't know if I have done things correctly but did you receive my work sheet about alcohol knowledge? I saved it but nothing happened after that!*	62 (6.6)	28 (49.1%)
Observes alcohol-related setback (inductive)	N/A	Text that expresses a setback (lapse) in relation to drinking goals or disappointment in relation to drinking	*I am really displeased with the Wednesday that I had four drinks, it's definitely a setback in relation to the goal.*	39 (4.1)	22 (38.6%)

### 3.3 Frequency and intercorrelations of client behaviors

The 57 clients sent a total of 472 messages, with a mean of 8.3 (*SD* = 5.6; range 2–30) messages. Within these messages, a total of 944 meaningful statements were coded into one of the 10 categories of client behaviors identified by Svartvatten et al. (2015) and 35 meaningful statements were coded into the inductive category *Observes alcohol-related setback*. As illustrated in Table 2, the relative frequencies of the clients' statements in the current study were as follows: *alliance* (26.6%; *M* = 4.25; *SD* = 3.62); *identifies patterns and problem behaviors* (22.8%; *M* = 3.51; *SD* = 4.36); *observes positive consequences* (7.2%; *M* = 1.16; *SD* = 1.95); *plans to attempt behavior change* (8.5%; *M* = 1.35; *SD* = 1.68); *reports on behavior change* (9.2%; *M* = 1.46; *SD* = 1.95); *avoidance of treatment* (5.5%; *M* = 0.82; *SD* = 1.28); *problems with technology and administration* (6.6%; *M* = 1.02; *SD* = 1.43); *problems with treatment content* (3.4%; *M* = 0.47; *SD* = 0.85); *maladaptive repetitive thinking* (5.7%; *M* = 0.88; *SD* = 2.11). *confrontational alliance rupture* (0.4%, *M* = 0.07; *SD* = 0.26); and *observes setback* (4.1%, *M* = 0.61; *SD* = 0.90). Intercorrelations among client behaviors showed several significant moderate to large correlations, with all behaviors correlating with at least one and as many as six other behaviors. *Alliance, identifies patterns and problems,* and *observes set-backs* were the most correlated behaviors; *avoidance of treatment* and *problems with treatment content* correlated with few other behaviors (see Supplement 1).

### 3.4 Correlations between client behaviors, outcomes and module completion

Table 3 describes correlations between the frequency of statements across all messages, the number of lessons completed and reductions in drinks and heavy drinking days at post-treatment and at 3-month follow-up.

**Table 3:** Correlations between client treatment-related behaviors, program adherence (module completion) and changes in drinking behaviors^a^.

Client behaviors	Program adherence	Post treatment outcomes		3-month follow up outcomes	
Category	Number of modules completed	Drinks preceding week	Heavy drinking days preceding week	Drinks preceding week	Heavy drinking days preceding week
Alliance	0.222	−0.003	0.007	0.133	0.082
Plans to attempt behavior change or use treatment exercise	0.147	−0.047	0.029	0.172	0.166
Reports on behavior change attempt or use of treatment exercise	0.213	−0.128	−0.081	−0.051	−0.044
Observes positive consequences of behavior change attempt or use of treatment exercise	0.217	−0.152	−0.205	0.028	−0.015
Identifies patterns and problem behaviors	0.225	0.052	0.125	0.219	0.142
Confrontational alliance rupture	−0.133	0.201	0.350⁎	0.066	0.133
Maladaptive thinking or anticipation of failure	0.050	0.183	0.225	0.261	0.228
Avoidance of treatment	−0.083	0.218	0.190	0.080	−0.008
Problems with treatment content	0.459⁎⁎	−0.116	0.023	−0.057	0.036
Problems with technology and administration	0.015	0.086	0.102	0.057	0.055
Observes setback	0.054	0.063	0.166	0.142	0.145
Number of modules completed	1	−0.365⁎⁎	−0.257	−0.119	−0.056

⁎ *p* < 0.05.

⁎⁎ *p* < 0.01.

a A positive correlation concerning drinking outcomes (drinks/heavy drinking days) indicates a given behavior was associated with symptom deterioration. A negative correlation here thus indicates a behavior was associated with symptom improvement.

Regarding program adherence, the number of modules completed had a significant positive correlation with *problems with treatment content* (*p* < 0.01). As for drinking outcomes, the number of modules completed was significantly associated with fewer drinks at post-treatment (*p* < 0.01), and *confrontational alliance rupture* was significantly associated with more heavy drinking days at post-treatment (*p* < 0.05). There were no other statistically significant correlations. The results were similar in the hierarchical regression-analysis (Table 4). Only *confrontational alliance rupture* (*p* < 0.05) and *problems with treatment content* (*p* < 0.05) were significant predictors of program adherence. Further, only *observes positive consequences of behavior change attempt or use of treatment exercise* (*p* < 0.05) and *confrontational alliance rupture* (*p* < 0.05) were significant predictors of heavy drinking days at follow-up, controlled for initial number of heavy drinking days. Similar to the correlational analysis, the number of modules completed significantly predicted number of drinks per week at post-treatment (*p* < 0.05) controlled for initial number of drinks per week, but did not predict any of the other drinking outcomes.

**Table 4:** Hierarchical regression analysis with client behaviors as potential predictors of outcome, controlled for the total number of client behaviors.

Client behaviors	Program adherence	Post treatment outcomes		3-month follow up outcomes	
Category	Number of modules completed	Drinks preceding week	Heavy drinking days preceding week	Drinks preceding week	Heavy drinking days preceding week
Alliance	0.013	0.007	0.014	0.008	0.001
Plans to attempt behavior change or use treatment exercise	0.001	0.004	0.001	0.004	0.011
Reports on behavior change attempt or use of treatment exercise	0.006	0.072	0.061	0.061	0.009
Observes positive consequences of behavior change attempt or use of treatment exercise	0.014	0.065	0.092* β = −0.385	0.016	0.018
Identifies patterns and problem behaviors	0.021	0.001	0.015	0.001	0.002
Confrontational alliance rupture	0.079* β = −0.298	0.019	0.099* β = 0.364	0.010	0.001
Maladaptive thinking or anticipation of failure	0.029	0.003	0.031	0.000	0.000
Avoidance of treatment	0.026	0.025	0.037	0.000	0.000
Problems with treatment content	0.104* β = 0.330	0.001	0.023	0.006	0.000
Problems with technology and administration	0.001	0.007	0.019	0.004	0.002
Observes setback	0.008	0.001	0.003	0.006	0.004
Number of modules completed	–	0.089* β = −0.298	0.052	0.007	0.000

∗ = *p* < 0.05, ∗∗ = *p* < 0.01.

Numbers represent the difference in explained variance (ΔR^2^) when adding the client behavior in question. Coefficients (β) are added for client behaviors with statistically significant differences in explained variance at the *p* < 0.05-level as a minimum.

### 3.5 Questions asked to therapists

Eighty-nine questions from clients to therapists were identified, and coded as belonging to the four categories in the previous study (Soucy et al., 2019), (see Table 5).

**Table 5:** Questions from clients to therapists.

Category	N (%)	Example quote
Questions about facilitating understanding and application of core techniques	36 (40.4)	Hi, what do you think about me having a non-alcoholic beer with dinner? It's not always so much fun with water together with a good meal.
Questions about therapy process	28 (31.5)	I have started with the exercises. Should I send them to you when the week is done, or should I send them several times as I fill them out?
Questions about technical issues	18 (20.2)	I don't know if I did things correctly, but did you receive my worksheet on alcohol education? I clicked on Save, but then nothing more happened!”
Questions about requesting assistance outside scope of program	7 (7.9)	In your reply, you wrote that if this treatment was not enough for me, there were other options. I hope that this treatment is right for me, but just in case, are there any alternatives that are as discreet if I should need them?

## 4 Discussion

This study investigated written messages from clients to therapists in a trial on internet interventions for AUD. We used the ten behavior categories identified by Svartvatten et al. (2015) after having made minor modifications in the coding manual in order to adapt these categories to the alcohol population. In addition to the previous ten categories, we also identified a new category specifically related to alcohol problems: *Observes alcohol-related setback*. The most frequently coded categories were *alliance* (26.6%) and *identifies patterns and problem behaviors* (22.8%), while the least frequently coded category was *confrontational alliance rupture* (0.4%). A significant correlation was found between *problems with treatment content* and module completion, such that more codings of problems with treatment content were associated with a larger number of modules completed. Another significant correlation was found between *confrontational alliance rupture* and heavy drinking days, such that more codings of *confrontational alliance rupture* were associated with a larger number of heavy drinking days post treatment. Also, we found a significant correlation between module completion and number of drinks, such that more modules completed were associated with fewer drinks at post-treatment. The additional regression analysis found similar results to the simple correlation with only a few significant associations linking client behaviors to adherence and outcome. Lastly, we identified the four main categories of questions that clients posed to therapists, as identified by Soucy et al. with proportions similar to those in the previous study (Soucy et al., 2019).

When comparing our results to the two previously published studies on client behaviors in internet interventions (Soucy et al., 2018; Svartvatten et al., 2015), several noteworthy observations stand out. In terms of the most commonly coded categories in our study, *alliance* was coded with a similar frequency in the study by Svartvatten et al. (22.3%), but with a higher frequency in the study by Soucy et al. (39.0%). In contrast, *identifies patterns and problem behaviors* were coded with a similar frequency in the Soucy study (25.1%), while the frequency was much lower in the Svartvatten study (6.4%). Findings thus suggest that while therapists who deliver internet interventions are likely to observe similar behaviors across internet interventions and populations, the frequency of such behaviors may vary greatly depending on the problem treated.

We found only one client behavior, *problems with treatment content*, that significantly correlated with module completion. It seems unlikely that having more frequent problems with the treatment content would cause a higher module completion. The reverse, that completing more modules increases the risk of the client being exposed to problems with treatment content appears more plausible at face value. Another possible explanation is that more motivated clients both adhered more and, in case of doubts on how to proceed, contacted the therapist more frequently. The Soucy study also only identified one category that significantly correlated with treatment completion, but in that case, the category was *tries alternative behavior* (i.e. *reports on behavior change attempt or use of treatment exercise* in our modified code manual). In the Svartvatten study, on the other hand, seven categories were significantly associated with module completion (Svartvatten et al., 2015), but none of these significant correlations were with the category identified in the current study (i.e. *problems with treatment content*). In terms of outcomes, we found only one significant correlation of a category with drinking outcomes, namely *confrontational alliance rupture*. Although this may intuitively seem like a clinically relevant finding, it should be noted that only four individuals were coded with *confrontational alliance rupture*, and the significant correlation was only observed with heavy drinking days post-treatment, not with drinks post-treatment. When compared with the other studies, *confrontational alliance rupture* was not significantly associated with negative outcomes in the Svartvatten study (Svartvatten et al., 2015), while in the Soucy study this category was not identified at all (Soucy et al., 2018). Svartvatten et al. (17) and Soucy et al. (18) identified other client behaviors that were related to outcomes on depression and anxiety (e.g., Soucy et al. found that *maladaptive repetitive thinking* was associated with smaller improvements in both anxiety and depression and *observes positive consequences* was associated with greater improvements in anxiety, while Svartvatten found that *alliance* and *observes positive consequences* were associated with improvements in depression). In the regression analysis, the category *observes positive consequences of behavior change attempt or use of treatment exercise* was a significant predictor of outcome, in addition to *confrontational alliance rupture*. As in the case with *confrontational alliance rupture* however, the category only predicted HDD post-treatment and not the other outcome measures. In sum, it is hard to see many similarities between our studies regarding correlations between frequency of categories and outcome. At this time, it does not seem advisable for therapists to make assumptions about client behaviors for predicting adherence or outcomes. Regarding the questions asked to therapists, these predominately focused on understanding and applying the core techniques, and on understanding the therapy process. It may therefore be important to incorporate answers to these kinds of questions when constructing new alcohol interventions, for example with Q&A sheets distributed at the start of treatment.

This study has a number of limitations. First, like the other studies mentioned above, the study is correlational, and therefore does not allow for causal inference. For example, although we found a significant correlation between *confrontational alliance rupture* and heavy drinking days post treatment, we do not know whether the alliance rupture caused an increase in heavy drinking days, or whether an increase in heavy drinking days caused the alliance rupture. Second, several categories were quite broadly defined, which might reduce their clinical utility. Third, since the study was exploratory, we did not control for the large number of correlations examined; the few significant findings that we found may for this reason be unreliable.

### 4.1 Implications and future research

Despite its limitations, this study represents a first step in elucidating client behaviors in internet interventions for AUD, and could help in developing a framework for understanding client behaviors in internet interventions for this population. This could be of value for clinicians when internet interventions are implemented in new clinical settings and help therapists understand what to expect and what they should be prepared to address (e.g., around 50% may report a problem with technology, 47% may report a behavior change or positive consequence; on the other hand, confrontational alliance ruptures are very rare). It is notable that 20% of clients randomly assigned to the guided internet intervention did not send more than one message to their therapist (and were thus excluded from the current study). This means that clinicians should anticipate that a significant portion of clients may not communicate with them at all. Although some significant correlations among categories and outcomes were identified in the current study, the sample size was low. Future studies could investigate whether there are overarching factors that might be more important in predicting response to treatment (e.g., maladaptive response, openness to treatment). Further, in the current study, the categories were based on cognitive behavior theory, as the treatment itself was based on this treatment model. However, there are other options for coding behavior. For example, in motivational interviewing (MI) (Molly Magill et al., 2015), theoretical concepts of change talk and sustain talk have been shown to be associated with drinking outcomes (M. Magill et al., 2018), and have been used to code client behaviors in studies on face-to-face interventions (Davis et al., 2016). Also, the interaction of client and therapist behaviors (i.e. sequential analysis) has been studied using MI concepts (Apodaca et al., 2016; Berman et al., 2019; Drage et al., 2019; Laws et al., 2018).

## 5 Conclusion

The current study may provide therapists with a better understanding of what to expect from clients who participate in internet interventions for AUD. Correlations between these behavior categories and drinking outcomes were not consistent, suggesting future studies should continue to explore strategies for categorizing client behaviors. It would also be valuable to understand therapist and intervention factors that may influence client behaviors. With currently ongoing initiatives for full integration of internet interventions within regular addiction services, the AUD population will have easier access to treatment than ever before. As this group is known to be reluctant to seek professional help, this development has potential to fundamentally change addiction services. Finding ways to optimize therapist guidance will be an important part of implementing AUD internet interventions in clinical settings.

The following is the supplementary data related to this article.

Supplement 1 Inter-correlations (Spearman's rho) among client behaviors. Supplement 1

## Declaration of competing interest

The authors declare that they have no known competing financial interests or personal relationships that could have appeared to influence the work reported in this paper.
