# Supplementary material for: Exploring client messages in a therapist-guided internet intervention for alcohol use disorders – A content analysis
Source: Internet Interv. 2021 Nov 11;26:100483. doi: 10.1016/j.invent.2021.100483 (PMC8605073; doi:10.1016/j.invent.2021.100483)
Supplement: Supplement 1 — Inter-correlations (Spearman's rho) among client behaviors. [file mmc1.docx]

**Supplement 1.** Inter-correlations (Spearman’s rho) among client behaviors

|  | 2 | 3 | 4 | 5 | 6 | 7 | 8 | 9 | 10 | 11 |
| --- | --- | --- | --- | --- | --- | --- | --- | --- | --- | --- |
| Alliance (1) | 0.46** | 0.27* | 0.51** | 0.64** | 0.11 | 0.19 | 0.23 | 0.13 | 0.27* | 0.38** |
| Plans to attempt behavior change or use treatment exercise (2) |  | 0.49** | 0.53** | 0.63** | -0.02 | 0.17 | 0.10 | -0.03 | -0.03 | 0.39** |
| Reports on behavior change attempt or use of treatment exercise (3) |  |  | 0.33* | 0.54** | 0.19 | 0.02 | -0.04 | 0.11 | -0.04 | 0.35** |
| Observes positive consequences of behavior change attempt or use of treatment exercise (4) |  |  |  | 0.44** | -0.15 | -0.06 | 0.02 | 0.03 | 0.02 | 0.22 |
| Identifies patterns and problem behaviors (5) |  |  |  |  | 0.12 | 0.36** | 0.16 | 0.17 | 0.14 | 0.48** |
| Confrontational alliance rupture (6) |  |  |  |  |  | 0.37** | 0.21 | 0.26* | 0.25 | 0.08 |
| Maladaptive thinking or anticipation of failure (7) |  |  |  |  |  |  | 0.23 | 0.14 | 0.31* | 0.52** |
| Avoidance of treatment (8) |  |  |  |  |  |  |  | 0.13 | 0.14 | 0.34** |
| Problems with treatment content (9) |  |  |  |  |  |  |  |  | 0.20 | -0.02 |
| Problems with technology and administration (10) |  |  |  |  |  |  |  |  |  | 0.10 |
| Observes setback (11) |  |  |  |  |  |  |  |  |  |  |

∗ = *p* < 0.05, ∗∗ = *p* < 0.01
